# Brief Measure for Screening Complicated Grief: Reliability and Discriminant Validity

**DOI:** 10.1371/journal.pone.0031209

**Published:** 2012-02-14

**Authors:** Masaya Ito, Satomi Nakajima, Daisuke Fujisawa, Mitsunori Miyashita, Yoshiharu Kim, M. Katherine Shear, Angela Ghesquiere, Melanie M. Wall

**Affiliations:** 1 National Institute of Mental Health, National Center of Neurology and Psychiatry, Tokyo, Japan; 2 Japan Society for the Promotion of Science, Tokyo, Japan; 3 Psycho-oncology Division, National Cancer Center Hospital East, Chiba, Japan; 4 Department of Palliative Nursing, Health Sciences, Tohoku University Graduate School of Medicine, Sendai, Japan; 5 Columbia University School of Social Work, New York, New York, United States; 6 Department of Psychiatry, Columbia University College of Physicians and Surgeons, New York, New York, United States of America; 7 Department of Biostatistics, Mailman School of Public Health, Columbia University, New York, New York, United States of America; Catholic University of Sacred Heart of Rome, Italy

## Abstract

**Background:**

Complicated grief, which is often under-recognized and under-treated, can lead to substantial impairment in functioning. The Brief Grief Questionnaire (BGQ) is a 5-item self-report or interview instrument for screening complicated grief. Although investigations with help-seeking samples suggest that the BGQ is valid and reliable, it has not been validated in a broader population.

**Methodology/Principal Findings:**

A questionnaire was mailed to a randomly selected sample (*n* = 5000) residing in one of 4 areas of Japan. The BCQ was examined for responders who were bereaved more than 6 months and less than 10 years (*n* = 915). Non-specific psychological distress was assessed with the K6 screening scale. Multiple group confirmatory factor analysis supported a uni-dimensional factor structure and the invariance of parameters across gender and age. Cronbach's alpha was sufficiently high (alpha = .75) to confirm internal consistency. Average Variance Extracted (0.39) was higher than the shared covariance (0.14) between BGQ and K6, suggesting discriminant validity.

**Conclusions:**

The results of this study support the reliability and validity of the BGQ in the Japanese population. Future studies should examine predictive validity by using structured interviews or more detailed scales for complicated grief.

## Introduction

Complicated grief (CG) is a bereavement reaction in which acute grief is unusually prolonged because of complications in the natural healing process. CG symptoms include intense yearning and sadness, preoccupation with thoughts of the deceased, excessive avoidance of reminders of the loss and rumination over circumstances or consequences of the death. Estimates of the prevalence of this condition among bereaved individuals range from 2.4% [1], to 6.7% [2]. In one study, prevalence of CG among older people who were actively grieving was 25% [3]. Bereaved individuals with complicated grief are thought to be at risk for co-occurring mental and physical health problems, including high mortality, cancer, heart trouble, high blood pressure, suicidal ideation, and changes in eating habits [4], [5]. Complicated grief has been proposed by the American Psychiatric Association's Diagnostic and Statistical Manual workgroup for inclusion in the 5th edition of the manual as a ‘bereavement related disorder’ [6]. The argument for DSM-5 inclusion was outlined in a recent paper [7].

Although it is widely known that complicated grief can lead to substantial impairment in quality of life, the condition is under-recognized [7]–[10]. Because normal, acute grief is also intensely painful, and because some complicated grief symptoms are similar to those of other disorders, such as major depression and post-traumatic stress disorder, accurate identification of people with complicated grief can be difficult for clinicians [11]. Furthermore, complicated grief often coexists with other disorders, most commonly depression and post-traumatic stress disorder [12]. However, even if a patient has a co-occurring disorder, complicated grief may still be the primary problem. Studies indicate that targeted treatment may be needed for complicated grief; accurate identification of symptoms is necessary to ensure that those with complicated grief receive effective treatment [13], [14].

Bereavement is universal and it is useful to have a very simple method of screening for complicated grief that can be used in clinical and research settings. The Brief Grief Questionnaire (BGQ) is a five-item scale that can be easily administered [15]. The scale was originally developed for a study of individuals who sought support following the 9–11 terrorist attacks in the New York City area. The scale showed good performance characteristics in that study. Further examination of the BGQ is, however, required for at least three reasons. Firstly, other studies that used the scale have been limited to high-risk populations who sought crisis or mental health services [13], [15]. Performance characteristics of the BGQ should be explored in a broader population. Second, there was no psychometric examination of the discriminant validity of BGQ. Previous studies reported the comorbidity of complicated grief and other disorders such as anxiety and depressive disorders [12], [15]. It is expected that BGQ would moderately correlate with anxiety or depressive symptoms, yet it should capture complicated grief distinctly from these symptoms. Third, an important factor to consider regarding complicated grief screening is the transcultural utility of clinical instruments. While social rules for expressing grief differ across cultures, the symptoms of complicated grief appear to be similar. However, because most previous findings were derived from a Western culture, study of the BCG in non-western cultures is important [1].

We used the same sample as a previous publication by Fujisawa et al. (2010) in the present study. This study found a prevalence of complicated grief of 2.4% among bereaved respondents from a randomly selected sample of individuals ages 40–79 residing in one of 4 areas in Japan, along with a 22.7% subthreshold prevalence of complicated grief. Furthermore, this study reported several risk factors for complicated grief: loss of a spouse, a loss that was unexpected, loss of a loved one due to stroke or cardiac disease, the death occurring at a hospice, care facility or at home, or the bereaved spent time with the deceased every day in the last week of their life. Although Fujisawa et al. (2010) used the BGQ as the measure of complicated grief, they did not report on the performance characteristics of the scale. The current paper uses the sample for which we previously reported prevalence findings. We now report on frequency distribution of the BGQ, its reliability, factorial validity, and discriminant validity.

## Methods

### Ethics Statement

The ethical and scientific validity of this study was approved by the institutional review board of the University of Tokyo. With the anonymous questionnaire, we enclosed a letter explaining the aim and informed consent procedure of this study. We regarded completion and return of the questionnaire as consent for participation in this study.

### Participants and Procedure

Individuals aged 40–79 years who had experienced the death of a loved one at least 6 months earlier participated in the present study, which is consistent with proposed criteria for complicated grief [7], [10]. Questionnaires (n = 5000) were mailed to randomly selected individuals aged 40–79 in four areas in Japan. These areas were selected to obtain a wide geographic distribution for the nationwide sample (Tokyo, the urban metropolis; Hiroshima, Miyagi, Shizuoka, the mixed urban-rural areas). We used a stratified two-stage random sampling method. First, we randomly selected 50 census tracts in each of the four areas. Then, we randomly selected 25 individuals in each census tract (*n* = 1250 per tract.) We mailed the study questionnaire in June 2009 and sent a reminder postcard 2 weeks later.

### Instruments

We used the BGQ to screen for symptoms of complicated grief [15]; see Appendix S1 and S2 for both English and Japanese versions. The scale consists of 5 questions about difficulty accepting the death, grief interference in current life, troubling thoughts related to the death, avoidance of reminders of the loss, and feeling distant from others. Each item is scored from 0 to 2 (0 = not at all, 1 = somewhat, 2 = a lot). We translated the BGQ into Japanese following the standard back-translation procedure. Briefly, two of the authors (MI, SN) translated the scale into Japanese, the translated version was back-translated by a bilingual Japanese clinical psychologist who did not know the original items on the BGQ, the back-translated version of BGQ was checked by the original author (KM) for concordance of meaning and expression, and after minor wording adjustments and modifications were completed, the original author (KM) confirmed the content validity of the back-translated version.

The K6 screening scale was used to assess non-specific psychological distress [16], [17]. The scale was originally developed for the U.S. National Health Survey to evaluate non-specific psychological distress, and is also used by the World Mental Health Survey. Notably, although often discussed as non-specific psychological distress, scale items describe common symptoms of depression and anxiety. The K6 items were derived from a pool of 612 questions taken from existing scales such as Beck Depression Inventory [18], Center for Epidemiologic studies – Depression scale [19], and State-Trait Anxiety Inventory [20]. Item-response theory analyses were used to select the most informative subgroup. Six items ask participants how frequently they in the last month experienced symptoms of depressed mood, anxiety, psychomotor agitation, worthlessness, hopelessness, and feelings of fatigue. Selected items performed well across gender, age, race/ethnicity, and educational and socio-demographic subsamples. The scale has been confirmed to have good performance in detecting individuals who meet criteria for mood and anxiety disorders in several countries [16], [17]. We used this scale to see whether the complicated grief assessed by BGQ is measuring a different construct.

### Statistical methods

BGQ characteristics were investigated by calculating item means and standard deviations. We examined frequency distributions of the total scale score and compared these to data obtained from the study of individuals who sought crisis services following the 9–11 terrorist attacks [15]. We also calculated mean and standard deviations of the BGQ total score for all participants and for subgroups of gender and age to determine differences between them. Cronbach's alpha coefficient and item-total correlations were examined to assess reliability.

Confirmatory factor analysis was conducted to examine the factorial validity of the BGQ. Based upon observations about complicated grief from earlier studies [10], [21], [22], we tested a uni-dimensional model. Further, we examined invariance of the model across four subsamples which were stratified by gender and age. Goodness-of-fit indices, including the root mean square error of approximation (RMSEA), the Comparative Fit Index (CFI), and the Tucker Lewis Index (TLI) were examined to indicate how well the model fit the data. Akaike's information criteria and chi-square difference tests were used to evaluate the relative fit of competing models. Standardized factor loadings were used to assess the appropriateness of the measurement for the latent factor.

Following the recommendation by Farrell and Rudd (2009), we examined the discriminant validity of BGQ from K6 in two ways. First, exploratory factor analysis for all items of BGQ and K6 was conducted to see whether the each scale items actually load on the assumed factors and does not cross-load on the different factors. In this exploratory factor analysis, we employed maximal likelihood method and oblique rotation for allowing the factors to be correlated. Next, we calculated the Average Variance Extracted (AVE) for BGQ and K6. This value is the average of squared each item's factor loadings. Because measurement error terms should be taken in account to correctly estimate the AVE, we performed the confirmatory factor analysis for BGQ and K6 as one model. If the AVE is greater than the shared variance (squared correlation coefficient) between the two factors, the discriminant validity is supported [23], [24]. All *p* values were two-tailed. The statistical packages PASW Statistics 17.0 (Chicago, Illinois) and AMOS 18.0 (Chicago, Illinois) were used for statistical analysis [25].

## Results

### Demographic characteristics and characteristics of bereavement

Of the 1970 questionnaires returned (response rate, 39.9%), 165 were excluded because of missing data for any of the following variables: gender, age, experience of loss in the past 10 years, time from bereavement, relationship with the deceased, and the items on BGQ. We excluded 775 respondents who had not experienced a loss in the past 10 years and 115 respondents who had experienced a loss less than 6 months previously. As a result, a total of 915 responses were subjected to analysis. Table 1 shows the demographic characteristics of the sample. Sixty percent were under 60 years of age and 58% were female. For participants who had experienced multiple loss, we instructed them to answer regarding the most recent death. The majority (73%) had lost a parent or a parent-in-law. About 25% were bereaved for 6 months–2 years, about 25% were bereaved for 2 years–4 years, and about half were bereaved for more than 4 years. The majority (about 60%) of the deaths occurred from cancer, stroke or cardiac causes, which is consistent with the results of vital statistics in Japan [26].

**Table 1:** Demographic data of the participants.

	*N*	%
Gender		
Male	384	42.0
Female	531	58.0
Age group (years)		
40–49	214	23.4
50–59	337	36.8
60–69	354	38.7
70–79	10	1.1
Relationship with the deceased		
Spouse	58	6.3
Parents	439	48.0
Parents-in-Law	232	25.4
Child	4	0.4
Sibling	87	9.5
Other	95	10.4
Time from bereavement		
6–12 months	106	11.6
1–2 years	136	14.9
2–3 years	131	14.3
3–4 years	112	12.2
4–5 years	84	9.2
5–6 years	79	8.6
6–7 years	94	10.3
7–8 years	57	6.2
8–9 years	47	5.1
9–10 years	69	7.5
Cause of death		
Cancer	335	36.6
Stroke	92	10.1
Cardiac disease	105	11.5
Other	381	41.6
Missing data	2	0.2

### Psychometric properties of the Brief Grief Questionnaire

**Figure 1:**
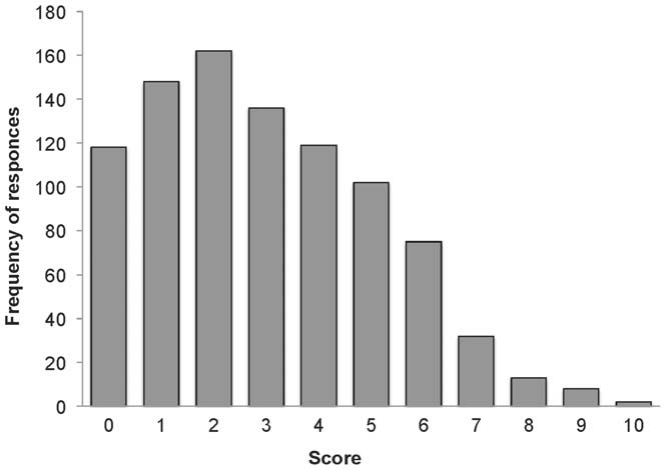
Distribution of total BGQ score (*n* = 915).

Table 2 shows the item means, standard deviations and the item-total correlations of the BGQ, and Figure 1 shows the distribution of BGQ scores across participants. The mean BGQ score in the entire sample was 2.99 (*SD* = 2.15) and the median was 3. The skewness and kurtosis were 0.51 and −0.37. BGQ total scores were significantly higher for females than males (mean (*SD*) for females = 3.17 (2.22), for males = 2.74 (2.04); *t* = 3.01, *p*<.01). There was no significant difference in mean scores between those over and under 60 years of age groups (mean (*SD*) for 40–59 years = 2.96 (2.16); 60–79 years = 3.03 (2.14); *t* = 0.46, *n.s.*). Item-total correlations were high (*rs*>.67) as was the internal consistency of the BGQ (Cronbach's alpha = .75), indicating adequate reliability.

**Table 2:** Item characteristics of the BGQ.

Item^a^	*M*	*SD*	Item-total correlation
Trouble accepting death	1.09	0.70	.70
Grief still interferes	0.45	0.59	.67
Thoughts that bother you	0.75	0.63	.67
Avoid doing things	0.31	0.52	.71
Feel cut off or distant	0.39	0.59	.76

a Response values were coded 0 = not at all, 1 = somewhat, 2 = a lot.

### Factorial Validity and Invariance

We tested whether parameters in the model varied across age and gender. The total sample was divided into four subgroups: *n* = 221 men aged 60–79 years ; *n* = 163 men aged 40–59 years; *n* = 330 women aged 60–79 years; and *n* = 201 women aged 40–59 years. Multiple group confirmatory analysis were performed to determine whether the parameters varied across different groups (Table 3). The structure of the three models was identical except in regard to the method of restriction. In model 1, we constrained all parameters including factor loadings and variance of error terms as well as the latent factor of complicated grief so that they were equal across all groups. In model 2, we assigned the factor loadings to be equal across all groups and the other parameters to vary between groups. In model 3, we set no restrictions on the model. Hence, model 1 is the most constrained and model 3 the least constrained. The chi-square tests between the model and AIC showed contradictory results. According to the chi-square tests, model 3 showed a better fit to the data than model 2 (χ^2^ = 24. 08, *p* = .020) and model 1 (χ^2^ = 54. 85, *p* = .004). In contrast, AIC was the smallest in model 1. Because all models showed good fit to the data (Table 3), invariance across age and gender can be assumed. Standardized factor loadings of each BGQ item on a single latent factor of complicated grief (model 1 in Table 3) were .65 (difficulty accepting the death), .74 (grief interference in current life), .73 (troubling thoughts related to the death), .55 (avoidance of reminders of the loss), and .38 (feeling distant from others). All parameters were statistically significant (*p*<.01).

**Table 3:** Goodness-of-fit indices for the different models and chi-square differences between the models.

Model	Goodness-of-fit indices for different models			Comparisons between groups			
	RMSEA(90% CI)	CFI	TLI	AIC	df	ΔChi^2^	*p*
Model 1^a^	.036 (.045; .027)	.943	.954	128.931	18	30.763	0.03^d^
Model 2^b^	.040 (.051; .029)	.955	.944	134.168	12	24.084	0.02^e^
Model 3^c^	.043 (.057; .030)	.967	.934	134.083			

RMSEA = root mean square error of approximation; 90% CI = 90% confidence interval of the RMSEA; CFI = comparative fit index; TLI = Tucker Lewis Index, AIC = Akaike Information Criterion, df = degrees of freedom.

a All parameters are assigned to be equal for all four groups.

b Factor loadings are assigned to be equal for all four groups.

c All parameters can vary between all four groups.

d Comparison between models 1 and 2.

e Comparison between models 2 and 3.

### Discriminant validity

**Figure 2:**
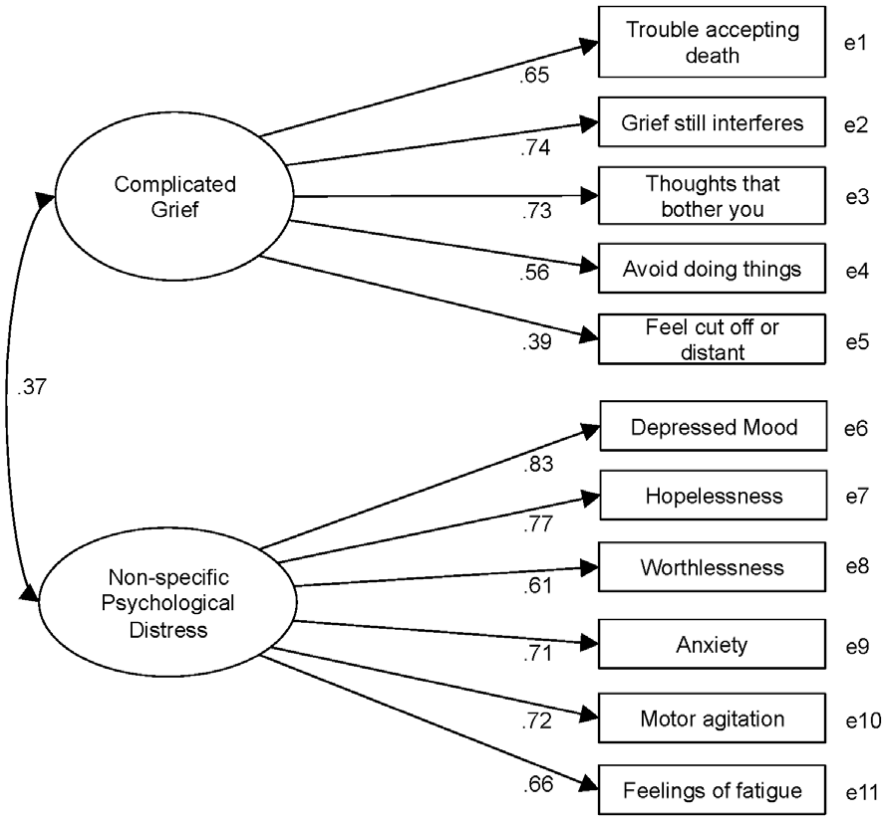
Confirmatory factor analysis of BGQ and K6.

Two eigenvalues of the exploratory factor analysis for all items of BGQ and K6 exceeded 1 (4.15, 1.97). All BGQ items loaded on the complicated grief factor (factor loadings>.33) and did not cross-load on the non-specific psychological distress factor (factor loadings<.11). Similarly, all K6 items loaded on the non-specific psychological distress factor (factor loadings>.63) and did not load on complicated grief factor (factor loadings<.08). Next, we tested the two-factor model of BGQ and K6 by confirmatory factor analyses (Figure 2). The goodness-of-fit indices of the model were sufficient (RMSEA = .052, CFI = 969, TLI = .952). AVE for BGQ and K6 were 0.39 and 0.51, respectively. The correlation coefficient between the latent factor of BGQ and K6 was 0.37. Hence, the shared variance between BGQ and K6 was 0.14. Both AVE for BGQ and K6 exceeded this value, suggesting discriminant validity of BGQ from non-specific psychological distress.

## Discussion

To the best of our knowledge, this is the first study that has investigated the reliability and validity of a screening questionnaire for complicated grief, in this case the BGQ, in a broad population. It is also the first study to examine validity and reliability of this scale outside of a Western country. Results confirm sufficient reliability, factorial validity and discriminant validity of the BGQ. These results could lead the cross-cultural conclusion that the BCG is a useful screening measure for CG that does not simply comprise a measure of general distress. Additionally, this study contributes to the body of information that strongly supports the inclusion of complicated grief as a new diagnosis in DSM5.

The reliability of the BGQ was suggested by several results. The internal consistency assessed by Cronbach's alpha was acceptable for the 5-item instrument, and the goodness-of-fit indices showed the validity of the uni-dimensional factor structure. This is concordant with the theoretical assumptions of this scale [15]. The fit-indices of the model were confirmed to be sufficient even when all parameters were assigned to be equal across different age and gender groups.

The modest correlation (0.37) between BGQ and K6 is consistent with previous studies showing complicated grief to be moderately associated with depression and anxiety [4], [15], [27], as well as PTSD and other anxiety disorders [12], [15]. While the BGQ and K6 were significantly correlated, the conceptual discriminant validity between these two variables was confirmed by multiple empirical tests. First, exploratory factor analyses showed the all items for each scale actually loaded on the assumed factor and did not cross-load each other. Then, AVE for each scale was higher than the shared variance between BGQ and K6, suggesting the discriminant validity [23], [24]. Furthermore, the sufficient goodness-of-fit indices provide support for the conceptualization of complicated grief and non-specific psychological distress as a distinct factor. Although many of who suffer from complicated grief show intense psychological distress of the type assessed by the K6 (e.g., depression, anxiety, and hopeless), the BCG is independently associated with functional impairment [15]. Additionally, complicated grief sometimes occurs without such comorbidity, even in a clinical help-seeking population [12]. These findings add to the existing literature that shows complicated grief to be a clinically significant condition that is not simply a manifestation of non-specific psychological distress which could be attributed to an existing DSM IV disorder.

Distribution of BGQ score differed from that of the previous study of 9–11 survivors who sought crisis counseling [15]. In our study, the distribution peaked with the tail to the right side whereas among those seeking crisis counseling, this tail was not seen. This difference is likely due to differences in the study samples. Whereas the current sample is population-based, participants in the previous study were highly selected. All were bereaved for 18 months or less by a dramatic and very violent act and sought crisis intervention services [15]. A traumatic or sudden death may be one of the risk factors for complicated grief [5]. Complicated grief is expected to be far more common among people seeking help than in the non-clinical population. Thus, the 2.4% rates of complicated grief reported by Fujisawa et al. (2010) for the current sample are clearly much closer to population prevalence than the 44.3% frequency reported by Shear et al. (2006). Perhaps of note, the distribution shown in the present study is similar to that for screening tools for other affective disorders.

### Limitations

Several limitations must be considered when interpreting our results. The response rate was not high (39.9%). It is possible that responders differ systematically from non-responders with respect to rates of complicated grief. Additionally, individuals experiencing more than one death were asked to focus on the most recent rather than the most difficult loss. Either of these factors may mean that the prevalence rates reported in Fujisawa et al. (2010) are too low. Data for this study were derived from a Japanese older adult population, and may not generalize to other countries or to younger populations. We did not collect information about religious beliefs or utilization of mental health services after bereavement, either of which could affect the grieving process. However, the purpose of this study was to understand BGQ performance in a non-Western sample. Results resemble those of studies conducted in the United States, suggesting that the BGQ performs similarly across cultures. In addition, most of the study participants lost parents. The rate of complicated grief is likely lower among those who lost a parent compared to those who lost a spouse or child. We did not include any other measure of complicated grief in order to provide convergent validity of the BGQ. However, in this study and in previous work with the BGQ, results are similar to those obtained with other complicated grief measures. Future studies should examine the predictive validity of the BGQ to detect complicated grief by using a more detailed scale or structured interviews to assess complicated grief.

### Conclusion

In summary, the findings of the present study suggest that the BGQ is a reliable and valid instrument for screening complicated grief in the non-clinical population of a non-Western country. The BGQ appears to be one of the few complicated grief screening instruments that has been examined in both clinical and non-clinical samples. The reliability and validity of the BGQ found in our Asian sample partly supports the notion of the cultural universality of complicated grief as a construct and the usefulness of the BGQ as a way to easily assess symptoms of complicated grief cross-culturally. Future study should examine the predictive validity of BGQ for widely used scales of complicated grief (i.e. the Inventory of Complicated Grief).

## Supporting Information

Appendix S1 **English version of Brief Grief Questionnaire.**
(TIFF)

Appendix S2 **Japanese version of Brief Grief Questionnaire.**
(TIFF)
